# Ligand Depletion *in vivo* Modulates the Dynamic Range and Cooperativity of Signal Transduction

**DOI:** 10.1371/journal.pone.0008449

**Published:** 2010-01-05

**Authors:** Stuart J. Edelstein, Melanie I. Stefan, Nicolas Le Novère

**Affiliations:** European Bioinformatics Institute, Hinxton, United Kingdom; Mount Sinai School of Medicine, United States of America

## Abstract

Biological signal transduction commonly involves cooperative interactions in the binding of ligands to their receptors. In many cases, ligand concentrations *in vivo* are close to the value of the dissociation constant of their receptors, resulting in the phenomenon of ligand depletion. Using examples based on rotational bias of bacterial flagellar motors and calcium binding to mammalian calmodulin, we show that ligand depletion diminishes cooperativity and broadens the dynamic range of sensitivity to the signaling ligand. As a result, the same signal transducer responds to different ranges of signal with various degrees of cooperativity according to its effective cellular concentration. Hence, results from *in vitro* dose-response analyses cannot be applied directly to understand signaling *in vivo*. Moreover, the receptor concentration is revealed to be a key element in controlling signal transduction and we propose that its modulation constitutes a new way of controlling sensitivity to signals. In addition, through an analysis of the allosteric enzyme aspartate transcarbamylase, we demonstrate that the classical Hill coefficient is not appropriate for characterizing the change in conformational state upon ligand binding to an oligomeric protein (equivalent to a dose-response curve), because it ignores the cooperativity of the conformational change for the corresponding equivalent monomers, which are generally characterized by a Hill coefficient 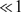. Therefore, we propose a new index of cooperativity based on the comparison of the properties of oligomers and their equivalent monomers.

## Introduction

Dose-response is one of the most common experimental approaches used by biologists to monitor the properties of signaling molecules. The power of this approach arises from the fact that the change in any quantifiable physiological response can be measured as a function of the chemical stimulus responsible. In some cases, the resulting curve is sigmoidal, which generally implies cooperative interactions between the binding sites for the ligand that initiates the response (but other explanations are possible — see below). In general, cooperativity (or ultrasensitivity) arises for numerous biological processes regulated by protein-protein or protein-ligand interactions involving multi-site proteins that transduce signals via conformational isomerization [1]–[3].

Cooperativity has been represented for numerous oligomeric protein systems by the allosteric model of concerted transitions [1]. The model is based on spontaneous transitions between two conformational states, designated 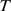 (for “tense”) and 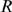 (for “relaxed”). The governing principle of the model is that in the absence of any bound ligands, the 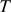 conformation is energetically favored over the 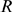 conformation. However, because the 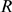 conformation has a higher affinity than the 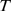 state for a ligand specific for the protein under consideration, the presence of ligand pulls the 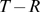 equilibrium towards the 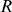 state. Under these conditions, a clear distinction can be made between two mathematical functions that describe the behavior of protein-ligand interactions as a function of ligand concentration: 1) the binding function, 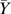, defined as the fractional occupancy of the ligand binding sites of the protein, taking into account both the 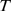 and 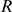 states; and 2) the state function, 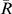, defined as the fraction of molecules in the 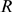 state. The state function 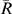 corresponds closely to what is measured by dose-response analysis for an allosteric “receptor” protein. The definitions of 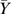, 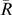, and various related parameters are summarized in Table 1.

**Table 1:** Summary of terms for cooperativity and ligand depletion.

Term	Description	Equation
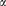	The concentration of ligand normalized to the affinity of the 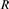 state	3
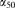	The value of 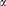 corresponding to 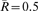	16
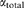	The value of 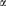 comprising both free and bound ligand	9
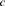	The ratio of ligand dissociation constants for the 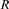 and 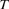 states	2
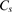	The molar concentration of ligand binding sites	9
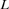	The allosteric constant governing the intrinsic 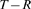 equilibrium	1
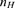	The Hill coefficient, defined by slope of loglog plot	18
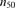	The Hill coefficient at 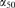	—
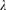	The allosteric constant governing the intrinsic equivalent monomers 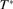 - 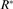 equilibrium	10
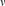	Cooperativity of the state function for an oligomer relative to the equivalent monomer	15
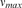	The maximal value of 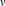 , which occurs at 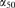	16
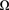	The ligand stabilization factor for 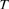 over 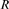	6
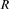	The “relaxed” (high affinity) conformational state	1
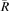	Fraction of total molecules ( 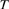 and 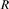 ) in the 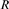 state as a function of 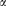	5
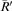	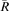 as a function of the total concentration ligand (free and bound)	9
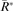	The fraction of equivalent monomers ( 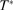 and 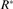 ) in the 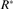 state	12
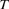	The “tense” (low affinity) conformational state	1
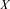	Any ligand	3
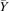	Fraction of all binding sites ( 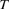 and 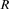 ) occupied by ligand	4
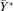	Fraction of equivalent monomer binding sites occupied by ligand	17
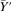	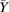 as a function of the total concentration of ligand (free and bound)	—

From its initial application to the sigmoidal oxygen-binding curve of hemoglobin, cooperativity has been conveniently characterized by the Hill coefficient, 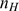 [4], [5]. The value of 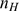 is obtained as the slope of the Hill plot: the logarithm of the ratio of occupied to unoccupied binding sites on the ordinate is given as a function of the logarithm of the ligand concentration on the abscissa. The value of 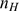 provides an empirical index of cooperativity: its upper limit is the number of interacting sites and its value is directly related to non-cooperative systems, since for a monomeric protein with a single site, 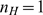. The Hill coefficient is widely used, including for dose-response curves, but care must be taken in interpreting its value [6]–[8], since kinetic effects can alter apparent cooperativity [9] and even a monomeric enzyme can display cooperative behavior, i.e. 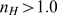 [10], [11].

Cooperativity can also be generated by relatively simple networks [12], for example through competition between two sets of phosphorylation sites [13], as well as sequestration effects involving an inactive complex [14] or more complex signal transduction cascades [15]. The interpretation of values of 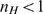, which can be a sign of negative cooperativity [16], also requires careful attention, since even for hemoglobin, binding curves with 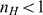 can be generated in the presence of non-stoichiometric concentrations of the positive effector, 2,3-diphosphoglycerate [17].

In addition to cooperativity, the non-linear properties of ultrasensitive systems define a dynamic range of signal intensities for which the responses vary. The greater the degree of cooperativity for a system with respect to signal changes, the narrower the dynamic range over which the response varies. For highly cooperative systems, such as bacterial chemotaxis, elaborate mechanisms have evolved in order to extent the dynamic range of response to changes in the concentrations of attractants or repellants [18], [19].

For all signal transduction systems considered, a predominant effect under physiological conditions is ligand depletion. When the concentrations of receptors are close to the dissociation constant for the relevant ligand, the free concentration of the ligand falls significantly below the total concentration of ligand, which in fact constitutes the actual input signal. This effect can be particularly important under *in vivo* conditions, for which most protein concentrations and dissociation constants are within the nano- to micro-molar range. The general principle of ligand depletion has been widely recognized [20]–[22] and various aspects have been considered for biological networks [14], [15], [23]. Here we focus on the consequences of ligand depletion with respect to cooperativity and dynamic range, as visualized for two extreme systems. First, we examine the highly cooperative flagellar motor system [24], [25]. Second, we turn to the minimally cooperative, but ubiquitous example of calmodulin [26], [27] in order to explore the consequences of ligand depletion under diverse conditions that apply in distinct regions of the brain and other organs. Finally, after illustrating why the Hill coefficient is not appropriate for measuring cooperativity of signal transduction, we define a new index of cooperativity, 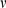, as illustrated with the classical example of the allosteric enzyme aspartate transcarbamylase [28], [29]. We show that 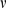, based on the introduction of an “equivalent monomer” concept, is a reliable measure of cooperativity for dose-response type curves under all conditions.

## Results

### Ligand Depletion and Dynamic Range in the Flagellar Motor System

We illustrate the importance of considering ligand depletion with the highly cooperative *E. coli* flagellar motor system [30], which controls the direction of flagellar rotation in response to the concentration of phosphorylated CheY [31]. The rotational bias of individual motors as a function of CheY-P has been measured using tethered single cells and GFP-CheY [30]. The motor bias reflects a change of rotation from counter-clockwise to clockwise and therefore a change of fractional activation (or state function, 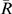), which is influenced by the interaction of CheY-P with the 34 units of FliM comprising the motor ring [32]. The data show a high degree of cooperativity, with Hill coefficients of up to 10 reported [30]. In contrast, the fractional occupancy, measured using FRET between CheY and FliM appears to be much less cooperative [31].

For dose-response measurements it is reasonable to assume equivalence to within experimental errors of the concentrations of the free and total ligand only if the protein to which the ligand is bound is present at sufficiently low concentration compared to the dissociation constant. However, for the measurements of the flagella motor system, free and total ligand were determined directly and were found to be far from equivalent [31]. The free concentration is significantly reduced compared to the total concentration, due to binding to FliM, as well as to CheA and CheZ [33]. In order to characterize this effect, we define 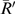, the response as a function of the total ligand concentration, which is distinct from 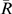, the response as a function of the free ligand concentration (see Table 1).

**Figure 1:**
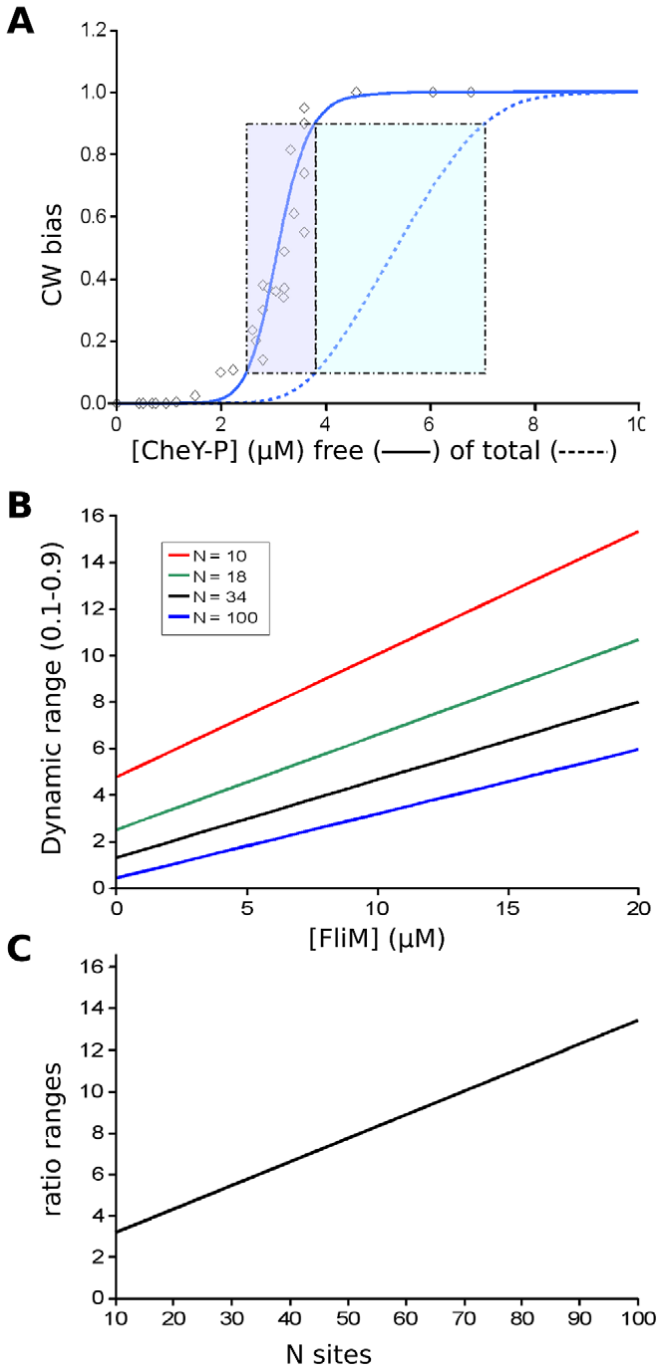
Flagellar motor model. (A) Curves for 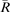 as a function of the concentration of free CheY-P (no ligand depletion: solid blue line) and curves for 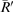 as a function of total CheY-P (with ligand depletion: dashed blue line), with 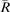 and 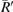 expressed in terms of CW bias, the measured parameter of the flagellar motor corresponding to the fraction of time undergoing clockwise rotation. The dynamic range, defined as the ligand concentration range between values of 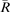 or 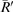 of 0.1 and 0.9, is represented by the shaded rectangles for the curves with and without ligand depletion. The open diamond points correspond to the measurements reported by Cluzel et al. [30]. (B) Variations in the dynamic range due to ligand depletion as a function of the concentration of FliM for values of 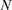 (the number of sites) = 10, 18, 34, and 100. For each value of 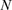, the curve for 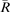 is computed based on an 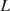 value set by 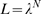 (see Materials and Methods, Eqn 11), where 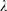 is fixed by the value used for 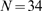, i.e. 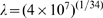. (C) The ratio of the dynamic range for 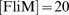
µM to the dynamic range for 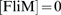 as a function of 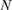, the number of sites and calculated as in (B). Parameter values used for the curves in (A): 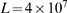, 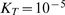 M, 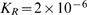 M, and 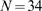, with a concentration of 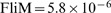 M. Calculation of ligand depletion effects as described in Eqn 9 of the Material and Methods section.

When ligand-depletion effects are taken into account, the curve for 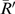 is displaced far to the right of the curve for 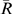 (Figure 1A). In addition, 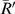 is significantly less steep than 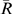. Moreover, the effect of ligand depletion on response curves is exhibited by all cooperative frameworks based on thermal equilibria, not only strictly concerted-models, such as proposed by Duke et al [34]. Therefore, ligand depletion results in an increase in the dynamic range of signal concentrations sensed by the system, as measured for instance by the differences in total concentration of CheY-P corresponding to 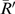 values between 0.1 and 0.9, which increase from 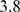
µM to 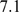
µM for a full change of response in this range. In comparison, the results presented using 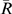 without taking into account ligand depletion could contribute to an underestimation of the dynamic range, since equivalent response changes would be achieved by increase from 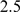
µM to 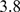
µM. With variations in the concentration of FliM, the dynamic range increases linearly (Figure 1B). More generally for multisite receptors, the dynamic range varies with the number of subunits, as observed for the family of curves in Figure 1B and 1C. Ligand depletion may also account for the discrepancies observed between the results reported by Cluzel et al [30] and other studies [35], [36] showing a much lower apparent cooperativity.

### The Effect of Ligand Depletion on the Response Characteristics of Calmodulin

**Figure 2:**
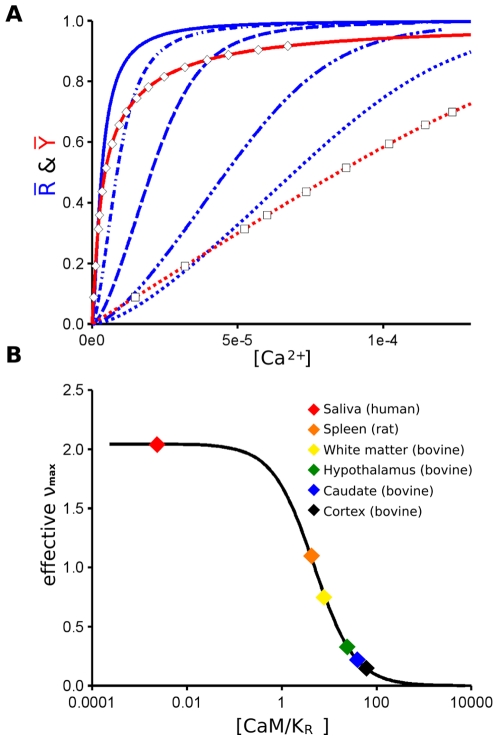
Ligand depletion for calmodulin. (A) Curves for 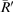 (blue) and 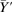 (red) as a function of the calcium concentration. (B) Values of effective cooperativity 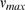 as a function of calmodulin (CaM) concentration/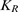, where 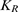 is the affinity of the 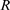 state for calcium. For the curves with solid lines in (A), 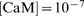 M and no ligand depletion occurs; the dashed curves for 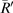 present conditions of ligand depletion based on the bovine brain calmodulin concentrations of white matter: 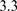
µM (- - - . . - - -); hypothalamus: 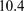
µM (- - - -); caudate nucleus: 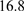
µM ( . . - . . ); and cortex: 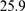
µM ( . . . . ), as reported by Kakiuchi et al. [40] or for 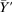 with the concentration of 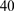
µM used in the measurements by Porumb [39], with data points shown as open squares. Although the calmodulin concentration of 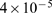 M [39] was close to the in vivo concentration of 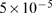 M in dendritic spines [60], the data were obtained by flow dialysis, which relates binding to the free calcium concentration, such that ligand depletion effects can be ignored, but we have transformed the data to simulate conditions of ligand depletion, with experimental points that closely follow the curve for 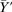, the fractional occupancy as a function of the total calcium concentration. The same calcium concentrations in (A) are used for the calculations in (B), with the addition of a value for saliva and rat spleen [40], [61]. Other parameter values as published previously [38] obtained using data from several sources. The curves under conditions of ligand depletion in (A) are calculated as described in the legend to Figure 1. Cooperativity in B is expressed in relation to the effective value of the index 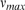 (Table 1), which decreases as a function of the total concentration of CaM.

In contrast to the behavior of a system of high cooperativity as described above, we examined the properties of calmodulin, a key molecule of calcium signaling with relatively low cooperativity [37], for which an analysis based on the MWC model has recently been presented [38]. The protein exists as a small monomer (148 residues), with four distinct calcium binding sites, each characterized by specific dissociation constants for calcium that vary between the low-affinity and high-affinity states [38]. Although the reference ligand binding properties that we used for our analysis are free of ligand-depletion effects [39], we have transformed the data to simulate conditions of ligand depletion, with points that fit the curve for 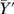 (the fractional occupancy as a function of the total calcium concentration) for calmodulin at 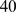
µM (Figure 2A). In addition, we have calculated a series of response curves presented in Figure 2A for the activation of calmodulin by calcium both under conditions with no ligand depletion (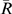), as well as under condition with ligand depletion (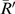) corresponding to various concentration of calmodulin found in vivo [40]. The differences between 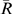 and 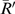 are very clear, including a progressive broadening of the dynamic range, with markedly diminished cooperativity as the concentration of calmodulin increases. The corresponding decreases in cooperativity as a function of calmodulin concentration are presented in Figure 2B, showing a dramatic fall off with concentration from the initial value 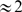 under conditions where Ca^2+^ is in large excess, to cooperativity values for the highest concentrations approaching zero.

### Limitations of the Hill Coefficient for Dose-Response Measurements and Introduction of a New Index Applied to Aspartate Transcarbamylase

**Figure 3:**
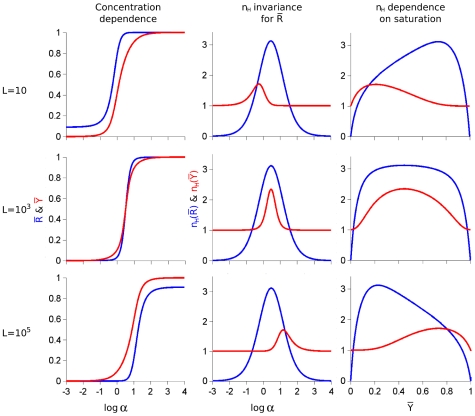
Dependence of 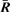 and 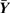 and their respective Hill coefficients (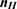) on the value of 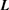. Three values of 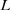 are illustrated, low 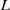 (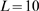; top three panels); intermediate 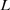 (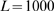; middle three panels — this value corresponds to the maximal cooperativity for the value of 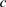 used: 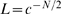, where 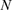 is the number of subunits or binding sites: 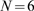); and high 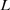 (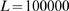; lower three panels). For each line of panels, the curves for 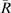 (blue) and 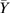 (red) are in the left panels, while the Hill coefficient (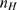) is presented as a function of 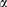 (middle panels) or of 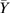 (right panels), in both cases for 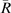 (blue) and 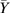 (red). The three panels of the central column illustrate that 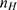 is invariant for 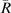 as function of 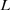. Therefore, as function of 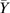 (three panels of the right column), the maximal value of 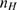 for 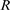 is at a high value of 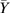 for low 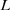 (upper right panel) and at a low value of 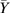 for high 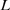 (lower right panel).

Since cooperativity of binding is generally evaluated by the Hill coefficient, 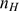, it is not surprising that the Hill coefficient has also been used to characterize many cooperative biological processes, including the fractional activation of signaling receptors and other proteins. However, as we shall demonstrate here, for conformational isomerization of a multi-site protein, 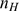 is not a reliable measure of cooperativity. In contrast to the cooperativity of 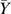, which varies with the energy difference of the two conformational states, as specified by the conformational isomerization constant, 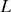, the value of 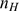 for 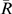 is independent of the value of 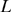 [41], as shown in Figure 3. When conditions of low, intermediate, and high affinity are examined for a hypothetical hexamer (Figure 3, left panels), the corresponding 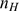 curves for cooperativity (Figure 3, middle panels) change appropriately for 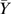, but are identical for 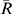 in the three cases. As a result, when cooperativity is examined as a function of 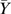 (Figure 3, right panels), the point of maximal cooperativity moves to the right for 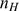 of 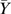 as affinity decreases, but the maximum value 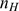 for 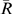 displays the opposite pattern.

**Figure 4:**
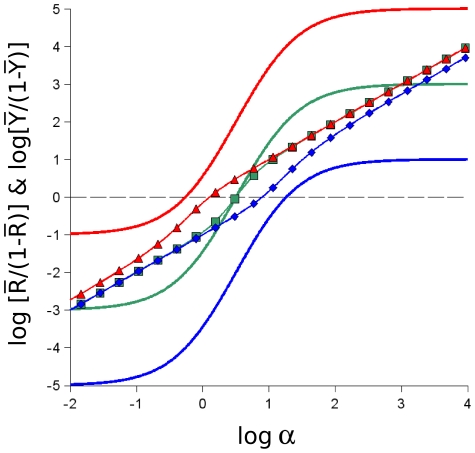
Hill plots for 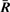 and 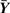. The data of Figure 3 (left column) are presented converted to the Hill plot, with the ordinate in the form of 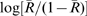 or 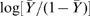. For the three values of 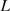 (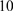, red curves; 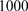, green curves; or 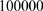, blue curves) the data for 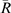 (solid lines) appear as parallel curves displaced vertically as a function of 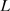. In contrast, the data for 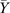 (triangles for 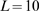, open squares for 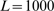, diamonds for 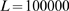) vary with the inflection points displaced progressively to the right with increasing magnitude of 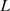.

Since 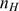 does not vary with the energetic difference of the two states, the shape of the curves for 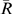 when expressed as Hill plots are invariant for different 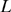 values, as shown in Figure 4. In contrast to the Hill plots of 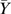, for which the shape changes as a function of 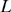 values, the curves for 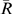 change only vertical position, not shape. Since cooperativity is generally measured around 50% response, correct results are obtained for 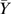, but the apparent cooperativity of 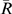 at 50%, i.e. 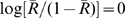 for a Hill plot, depends on the vertical position of the curve for 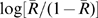 and is only a valid estimate of cooperativity for 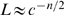 (Figure 4, green curve). The differences in shape between the curves for 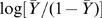 and 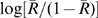 also explain why the cooperativity curves in Figure 3 (middle panels) tend towards 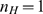 at the extremes for 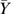, but towards 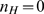 at the extremes for 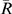. Values of 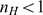 are commonly considered to be characteristic of negative cooperativity rather than the absence of cooperativity, but the properties of 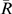 curves represent a special case for which the conventional reasoning does not apply. Overall, the analyses presented in Figures 3 and 4 make clear that as a general parameter to characterize 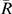 under any conditions, the Hill coefficient is not a reliable measure of cooperativity.

**Figure 5:**
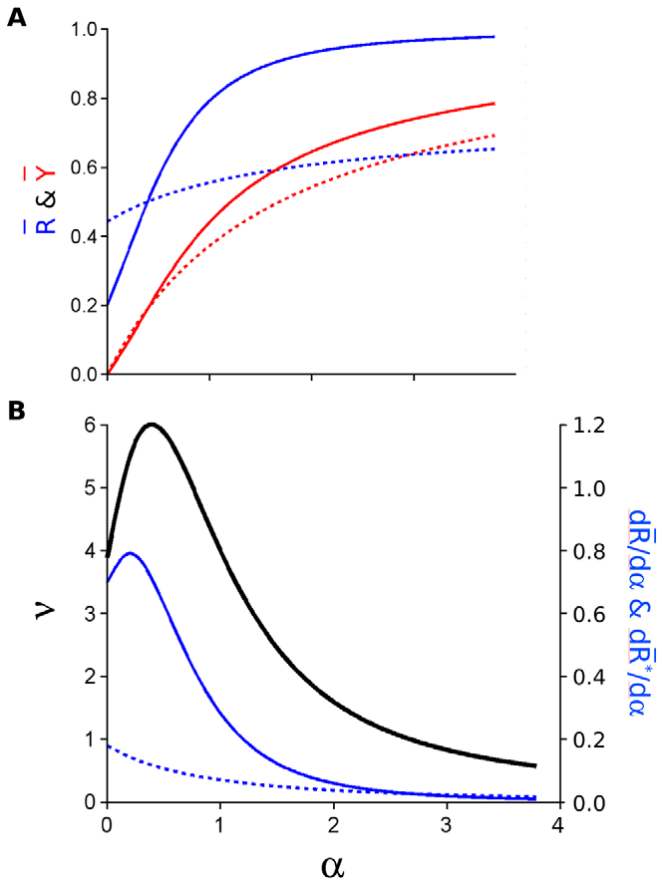
New measure of cooperativity for aspartate transcarbamylase based on an equivalent monomer. (A) Curves for 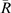 and 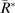 (in blue) and 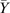 and 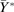 (in red) as a function of 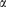 (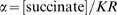); the curves for 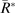 and 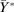 are dashed. (B) Values of 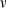 in black corresponding to the left ordinate and values of the derivatives 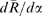 and 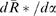 in blue corresponding to the right ordinate, with the latter as a dashed curve. While curves for 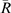 and 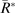 in A cross at 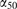 (defined by 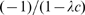, with a value 0.5 (see Material and Methods, Eqn 16) at this point, the curves for 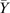 and 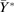 also cross at 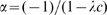, but their value is 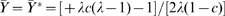, which only equals 0.5 for 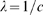. For the conditions presented here, at the cross point: 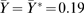. The original analysis based on the MWC model with four subunits used the values of 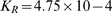 M, 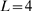 and 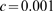 [45]. The model was re-analyzed by generating theoretical curves with the original parameters for a tetramer and performing a least-squares fit to obtain the best parameters for the hexamer, resulting in a change of the value of c to 0.26, when 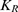 and 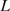 were unchanged. For ATCase, ligand depletion was not considered, since experimental results were obtained at concentrations of the enzyme for which ligand depletion was negligible and even in overproducing strains [62] ligand depletion is only a minor effect *in vivo*.

In order to overcome the limitations of the Hill coefficient applied to 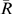, we reexamined how cooperativity is computed for conformational isomerization using data for the allosteric enzyme aspartate transcarbamylase (ATCase), one of the original examples of allosteric phenomenon [42]. Following the formulation of the two-state MWC model [1], it was recognized that under many conditions, 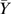 and 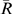 as a function of ligand concentration would not overlap [43]. In a classic study of ATCase, the direct binding of succinate (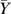) was compared to the succinate-dependent conformational change (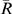) as measured by sedimentation or reactivity of protein sulfydryl groups [44], [45]. ATCase was initially characterized as a tetramer, but later studies revealed a hexamer [46], [47] and subsequent structural studies have thoroughly characterized the two hexameric conformational states, T and R, and their concerted interconversion [48], [49]. Using the parameters of the MWC model established for 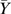 and 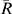 data on the basis of four sites, the theoretical curves were recalculated with six sites, as presented in Figure 5. Under the experimental conditions employed, the curve for 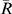 is substantially to the left of the curve for 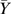, which constituted strong evidence a conformational equilibrium pre-existing to ligand binding [45]. When the Hill coefficients are determined at 50% for both the 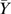 and 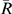 curves, the value of 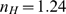 for 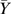 is a reliable measure of the cooperativity, but the value of 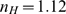 for 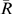 dramatically underestimates the intrinsic cooperativity, as we now demonstrate.

In order to establish the correct intrinsic cooperativity of an oligomeric protein undergoing conformational isomerization, a reference state is required that corresponds to a hypothetical equivalent monomer, characterized by same intrinsic affinities for ligand of the 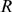 and 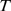 states. A conformational transition of the equivalent monomer as a function of the binding of its ligand can be defined and is represented here by 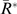, along with the binding to the equivalent monomer represented by 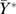. For an equivalent monomer, the energy difference between the 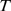 and 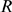 states is postulated to be 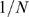 of the energy for the oligomer, since the energy difference for the oligomer is spread equally over the 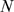 subunits. Therefore, we define 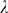, a conformational isomerization parameter for the equivalent monomer, where 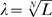 (see also Materials and Methods, Eqn 11). When the curves for 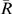 and 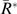 are compared as in Figure 5A, they cross at the value of 0.5 (which is true for all symmetrical MWC-type systems), but the curve for the equivalent monomer is clearly much more shallow.

**Figure 6:**
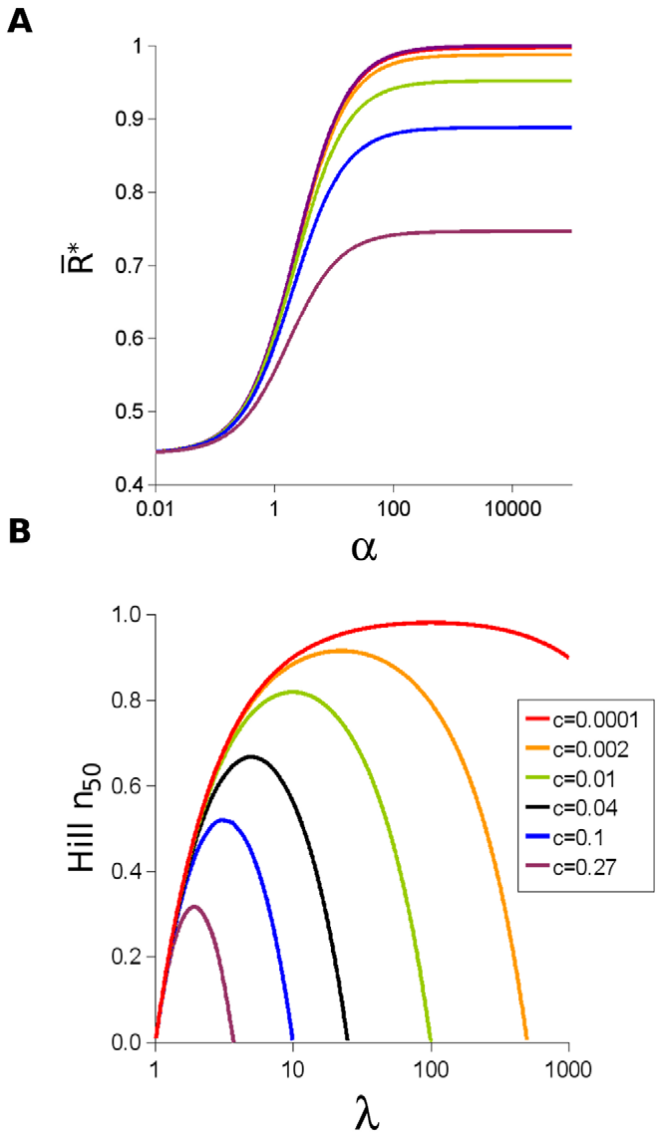
Properties of equivalent monomers. (A) Dependence of the state function 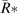 versus 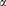 on the value of 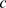. Six values of 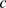 are presented corresponding to the color code indicated in the inset to the figure. (B) Value 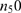, the Hill coefficient at 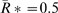, as a function of the monomer transition parameter 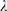 for the six values of 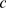 presented in (A) with the same color code.

With respect to ligand binding, the curves for 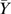 and 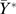 in Figure 5A differ only slightly and are characterized by Hill coefficients of 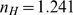 and 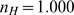, respectively. In contrast, the 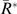 curve, with a Hill coefficient of 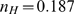 is much less cooperative than the curve for 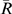, with 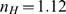, exactly 6-fold higher than the value for 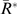. In general, under virtually all conditions 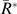 is characterized by a value of the Hill coefficient, 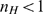 (see Figure 6).

In order to overcome the insensitivity of 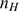 for 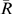 to 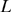 (Figure 3) and to rely on an appropriate reference state corresponding to the equivalent monomer, we propose replacing the Hill coefficient for dose-response type behavior by a new cooperativity index, 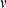 (Greek letter nu), based on the ratio of the derivatives of the functions for 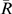 and 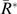. The function 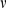 therefore corresponds to the ratio of the slopes exhibited by the responses of the cooperative protein and its equivalent non-cooperative monomer. When the new derivative functions are calculated, for 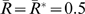 for the ATCase data in Figure 5, the values of the derivatives are 0.710 and 0.118, respectively, with a ratio of 6.0. The new cooperativity index 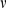 can also be computed directly from the definition of 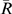 and 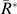 (see Material and Methods, Eqn 15). For ATCase, direct calculation also yields 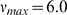.

The revised analysis of ATCase illustrates that the intrinsic cooperativity at 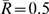 is always maximal, i.e. equal to the number of binding sites (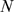), when compared to the equivalent monomer reference state for symmetrical oligomeric proteins. In other words, for a multi-site protein that undergoes a concerted conformational transition, as defined by the MWC model [1], the maximal cooperativity is always equivalent to the number of ligand-binding sites present and may be grossly underestimated on the basis of the Hill coefficient. This property reflects the absolute linkage, or infinite junctional energy, between binding sites in the MWC framework [34]. When data for the flagellar motor is re-examined in this context, the ratio of the derivatives of 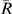 and 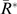 at 50% (Figure 1) corresponds precisely to the value of 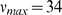. The value of 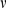 represents the intrinsic cooperativity of the protein and 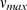 is not affected by ligand-depletion. For various signal transduction systems, the intrinsic cooperativity can, however, be modulated by ligand depletion effects. In order to characterize the effects of ligand depletion on cooperativity we calculated the effective 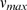 by correcting 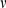 for the ratio of the slopes of 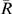 and 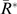 for corresponding fractional activations. As shown for calmodulin (Figure 2B), as for any sensor protein that possesses intrinsic cooperativity, ligand depletion can dramatically reduce the effective cooperativity in a physiological context. Indeed, this effect can bring the effective cooperativity to near 0 (Figure 2B). Because of non-equivalence of the four calmodulin ligand-binding sites, the non-identical dissociation constants for the sites result in the value of 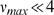 in Figure 2B.

## Discussion

Since many cellular control networks involve cooperative interactions among their components, modeling in the context of complete systems requires accurate estimations of the cooperativity of individual reactions. Since ligand depletion can exert an attenuating effect on cooperativity, it is important to have reliable estimates in the absence of ligand depletion. As illustrated in Figure 3, the Hill coefficient as applied to the state function of the MWC model, 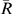 (equivalent to a dose-response curve) clearly does not reflect the correct cooperativity of the response, due to the invariance in the shape, as visualized in the Hill plot presented in Figure 4. As a result, when applied to the classical allosteric enzyme, aspartate transcarbamylase, the difference between the functions for ligand binding (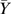) and change of conformational state (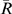) are not meaningfully characterized by their respective Hill coefficients. The value of 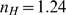 for (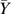) accurately reflects the correct degree of cooperative binding, since it contrasts with the non-cooperative case, with 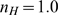. In comparison, for 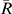 the observed value of 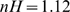 is not meaningful, since the non-cooperative case, as expressed by the corresponding “equivalent monomer,” displays a value of 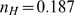. The correct extent of cooperativity of 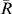 can be calculated from the ratio of these two values, or directly from the new index, 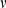, as defined in Eqn 15, with 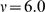 in the case of 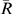 for ATCase.

The results presented here demonstrate that neither dynamic range nor effective cooperativity are properties of sensing proteins that can be considered to be invariant; rather than are likely to vary according to the organ, tissue, or cell-type. The concentrations of most signaling proteins are similar to their dissociation constants, in the nano- to micro-molar range, as for example in the well-characterized compartment of the PSD signaling complex of dendritic spines [50]. For calmodulin, it is particularly clear that ligand-depletion is common under physiological conditions, as shown in Figure 2, with the exact consequences depending on the tissue. Related examples include the interaction of calmodulin with other downstream components, such as calcineurin in the micromolar range [51]. While dose-response curves provide the basic characterizations of “systems” and therefore lie at the core of pharmacological treatments, in the analyses presented here we show that dose-response parameters cannot be reused directly in models of signaling systems. Instead one needs to build “mechanistic” models and run parameter-fitting approaches for particular conditions. Although we emphasized the effects of ligand depletion using the allosteric model [1], the general conclusions would apply equally well to other mechanistic descriptions, including the classical Adair-Klotz formulation [52].

It is also important to emphasize that cooperativity and dynamic range can change with the level of expression of the sensor. It is known that the available pools of signaling proteins can be quickly modified by segregation, inhibition, or change in expression. Because of the extreme cooperativity of the flagellar motor, ligand depletion dramatically increases the dynamic range of the system, as shown in Figure 1, making this system extremely sensitive to concentration effects. Since flagellar protein concentration will ultimate influence these properties, it is therefore clear that by changing the number of motors, bacterial cells could enhance their adaptation properties. Since the number of flagella per bacterial cell can vary considerably [53], this parameter must be taken into account for any complete characterization of chemotaxis [54]. More generally, the use of ligand depletion could be a widespread physiological mechanism for cells to adapt non-linear properties and sensitivity ranges to evolving environmental conditions. Because ligand depletion can decrease the effective cooperativity of transducers *in situ* and increases the dynamic range, we propose that modifying the concentration of the sensor may be a powerful way to adapt quickly to a new environment and switch from a measurement mode to a detection mode.

As modeling of biological phenomena encompasses systems of increasing complexity, particularly in efforts to develop realistic models of the nervous system [55]–[59], it is important to represent the underlying molecular processes as accurately as possible. The results presented here, in line with other published findings [14], [15], [20]–[23], emphasize that cooperativity and its consequences, especially dynamic range, cannot be introduced into models as fixed parameters based on Hill coefficients estimated from *in vitro* studies. Rather, each set of reaction components must be evaluated separately with respect to effects of concentration in the system examined, in order to describe accurately the functional properties that apply.

## Materials and Methods

### Dose-Response Relationships for an Oligomeric Protein with Two Conformational States

We consider a multisite signaling protein that can interconvert between two functionally distinct conformational states, a more active state (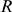) with a high affinity for ligand (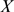) and a less active state (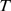), with a low affinity for the ligand. The partition between the two states is characterized by 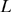, the relative intrinsic stability of the two states in the absence of ligand:
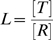
(1)


The affinities of the 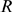 and 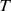 states for the specifically bound ligand are characterized by the intrinsic dissociation constants: 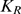 and 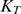. For convenience, as originally proposed in the MWC model [1], the ratio of affinities is represented by 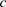:
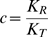
(2)and the parameter 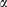 is defined as the normalized ligand concentration:
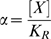
(3)


Using these parameters [1], for a protein with 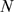 sites, the binding function is given by:
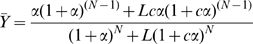
(4)and the state function is given by:
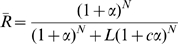
(5)


In order to generalize Eqn 5 to multiple ligands, we introduce a new parameter, 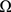, to describe the relative stabilization of the T state by a ligand:
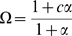
(6)


For a protein with 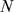 sites, at any concentration of 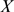, the state function 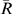 is then given with respect to 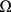 by:
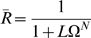
(7)


For 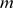 different ligands binding on multiple sites to the same protein, 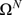 in the above equation is replaced by the product of 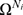 for the respective ligands:
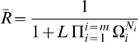
(8)


Since 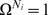 if the number of sites is 0, the concentration of the effector is 0, or the affinities for the 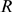 and 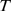 states are identical, this formula actually describes the absolute state function, modulated by any possible effector [43].

### Calculation of Ligand Depletion

Under conditions of significant ligand depletion, i.e. ligand concentrations in the same range as dissociation constant, the degree of ligand binding to its receptor cannot be calculated directly from the total concentration, because only a fraction of this concentration is “free” and available to participate in the binding equilibrium. For any total concentration, the corresponding free concentration can be calculated with respect to a given receptor concentration as one of the roots of the appropriate second-order equation [22]. However, a simpler approach was used here. We define the parameter 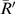 to define 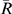 as a function of the total concentration. For each value of 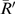, the corresponding value of the total concentration, expressed as 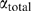 total, is calculated from the equation:
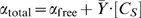
(9)where 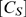 is the concentration of ligand binding sites. Multiplying 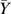 by 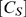 therefore provides a correction factor that when added to 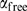 gives 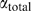.

### The Index of Cooperativity, 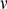, for an Oligomer with Respect to Its Equivalent Monomer

In order to evaluate the cooperativity of 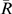 versus 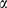, it must be compared to the properties of a single-site “equivalent monomer.” For any conditions of 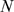, 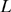, and 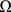, we postulate an equivalent monomer with transitions between monomeric states 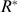 and 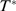 defined by:
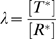
(10)where
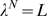
(11)


For a symmetrical system composed of identical ligand-binding sites, the fraction of monomers in the 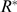 state is given by:
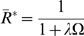
(12)


In this case, the curves for 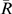 and 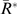 as a function of 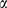 cross at 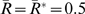. The slopes of 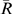 and 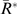 versus 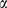 are obtained from, respectively, the following derivatives:
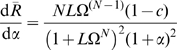
(13)and
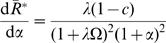
(14)


The intrinsic cooperativity or amplification of the signal reflected by the properties of 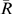 can then be obtained by a new parameter, represented by the coefficient 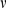 (the Greek letter nu) and calculated from the ratio of the two derivatives above (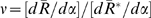) which simplifies to the equation:
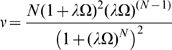
(15)


The coefficient 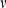 gives the cooperativity of the oligomeric protein for the state function 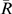 in a manner analogous to 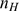 (the Hill coefficient) for the binding function (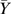), which describes cooperativity with respect to a monomer that in every case displays a value of 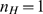. In contrast, when applied to 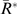, the Hill coefficient is likely to be substantially less than 1 (see Figure 6B), demonstrating why the Hill coefficient is inappropriate for estimating the cooperativity of 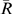. For a given value of 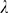 the lower limit of 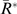 is given by 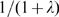 and the upper limit of 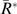 is given by 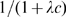, with the curves for 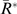 as a function of 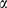 described in Figure 6A. The intersection of the curves for 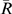 and 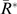 at 0.5 corresponds to the value of 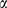 defined as 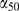 and is given by:
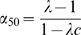
(16)


Under these conditions, 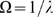 and the 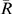 cooperativity parameter is at its maximal value: 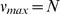 (whereas 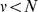 for all other values of 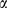).

### Derivation of the Hill Coefficient for an Equivalent Monomer

With respect to ligand binding, compared to Eqn 4 for fractional ligand binding, 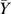, within the context of the two state MWC model [1], the corresponding equation for fractional binding to the equivalent monomer, 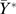, is given by:
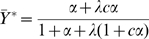
(17)


The Hill coefficient, 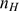, is defined by the derivative:
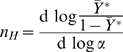
(18)


Substituting Eqn 17 for 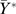 yields:
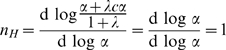
(19)


In contrast, 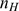 for 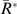 as defined by Eqn 12 yields the derivative:
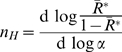
(20)


Substituting Eqn 12 for 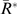 yields:
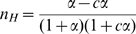
(21)


Therefore, since 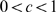 and 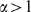, it is clear that:
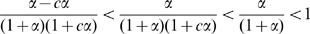
(22)and hence for 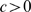, the Hill coefficient for 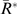 must be 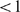 (additional details in M. Stefan, Thesis, University of Cambridge, 2009).
